# Assessment of Physical Activity Indicators for Children and Youth in Ethiopia: Evidence from the Global Matrix 3.0 Study (2017–2018)

**DOI:** 10.1186/s40798-019-0229-5

**Published:** 2019-12-30

**Authors:** Chalchisa Abdeta, Zelalem Teklemariam, Alem Deksisa, Endashew Abera, Reginald Ocansey, Anthony D. Okely

**Affiliations:** 1 Department of Physiotherapy, Hiwot Fana Specialized University Hospital, Harar, Ethiopia; 2 College of Health and Medical Sciences, Haramaya University, Harar, Ethiopia; 3 Department of Public Health, Adama Hospital Medical College, Adama, Ethiopia; 4 Clubfoot Program, Cure Ethiopia Children Hospital, Addis Ababa, Ethiopia; 5 School of Education and Leadership, University of Ghana, Accra, Ghana; 6 Early start, Faculty of Social Science, University of Wollongong, Wollongong, Australia

**Keywords:** Physical activity indicators, Sedentary behavior, Children, Ethiopia

## Abstract

**Background:**

Regular physical activity is vital for children’s health, well-being, and development. However, evidence is scant about physical activity indicators for children and youth in Ethiopia. This study aimed to assess physical activity indicators among children and youth in Ethiopia.

**Methods:**

This study was conducted as part of the Active Healthy Kids Global Alliance’s “Global Matrix 3.0” which included 49 countries. Data were collected from December 2017 to April 2018. The country research team included different disciplines related to physical activity. Data were retrieved from pre-reviewed literature, government policy documents, and an expert interview panel. Data were analyzed using the ten physical activity indicators for children and youth. The grading system was done through a harmonized process and the standard grading rubric of the Global Matrix 3.0 study ((A = ≥ 80%, B = 60%–79%, C = 40%–59%, D = 20%–39%, F = < 20%, INC = incomplete data).

**Results:**

For the overall physical activity indicator, 28% of children and youth in Ethiopia met the recommended physical activity of 60 min per day which resulted in a “D” grade. Likewise, the school and government indicator received a “D” grade. Almost 32% of schools in Ethiopia had access to infrastructures and multipurpose spaces for physical activity including outdoor play. The government policy partially existed in the non-communicable diseases agenda but had less focus on children and youth. The active play indicator scored the highest grade of “B.” About 71% of children and youth were involved in active play for at least 2 h a day before, during, and after school. About 50% of children and youth were engaging in organized sport participation, and this indicator was graded a “C.” Similarly, 48% of children and youth walked to and from school as a means of active transportation resulting in a “C” for this indicator. Three indicators (sedentary behavior, family and peers, and community and environment) were graded as an “F.” Approximately 8% of children and youth were living in communities and environments that did not support opportunities for physical activity. Only 13% of children and youth spent less than 2 h per day in sedentary screen time. There was no adequate information to grade the physical fitness indicator.

**Conclusion:**

This study showed that Ethiopian’s children and youth have received low grades for majority of physical activity indicators. Therefore, urgent actions should be taken by the government, policymakers, researchers, and key stakeholders to address the suggested priority areas.

## Key Points

This study lays a foundation for future study on physical activity policy, practice, research, and surveillance among children and youth in Ethiopia. The following three points should be prioritized to improve the current situation:

- Develop and implement a consistent national physical activity research and surveillance system in the country.
- Formulate physical activity policy with sufficient resource allocation for physical activity interventions.
- Develop, implement, and evaluate a National Physical Activity Plan for children and youth

## Background

Physical activity can be defined as any movement made through our body that raises energy expenditure above basal levels [1, 2]. It can comprise structured or unstructured activities providing many opportunities for children and youth to engage [2]. Regular physical activity is vital for children’s health, well-being, and development. Evidence shows that physical activity is important for children’s physical, physiological, mental, and intellectual development [3–6].

The World Health Organization (WHO) recommends that children and youth aged from 5 to 17 years old engage in a minimum of 60 min of moderate- to vigorous-intensity aerobic activity on a daily basis [7]. To meet this recommendation, children and youth can be engaged in any form of physical activity including active play, home-/school-based body movements, walk or bike, and structured exercises [1, 7].

Physical inactivity is defined as not meeting the recommended level of physical activity [7]. Current evidence suggests that about 80% of the world’s school-aged adolescents (11–17 years) are physically inactive [8, 9]. Ethiopian children are in a lifestyle shift toward physical inactivity [10–12]. Evidence suggests that childhood inactivity has contributed to the rise in obesity in many developing countries including Ethiopia [13]. A systematic review conducted on overweight/obesity among children and adolescents in Ethiopia found that the combined prevalence of overweight and obesity among children was 11.3% (overweight = 8.9%, obesity = 2.4%). Factors such as female gender, higher income, and physical inactivity were linked to the prevalence of overweight/obesity [14].

Sedentary behavior is any waking activity in a sitting or lying posture that is less that expends less than 1.5 metabolic equivalents (METs) [15, 16]. Children spend most of their time at home/school sedentary. This includes large amounts of time using sedentary screen-based technologies such as smartphones, computers, television, and electronic gaming consoles [16]. A low level of physical activity and high levels of sedentary behavior among children and youth increases the risk for non-communicable diseases in adult age [16–19]. The Active Healthy Kids Global Alliance (AHKGA) (www.activehealthykids.org) was established since 2014 as a not-for-profit organization. The AHKGA is working with researchers, professionals, and key stakeholders in the area of physical activity and sedentary behavior among children and youth. The AHKGA has developed a Report Card for participating countries. To date, the AHKGA has released Global Matrices 1.0, 2.0, and 3.0 in 2014, 2016, and 2018, with 15, 38, and 49 countries, respectively, having participated in each of these years [20].

Ethiopia participated for the first time in the Global Matrix 3.0 study. Ethiopia is located in the horn of Africa with an estimated population of 102 million in 2016, making it the second most populous nation in Africa. Of this population, 42.6 million (41.6%) are children and youth under 15 years of age [21]. Ethiopia is classified as a low-income country with a Human Development Index (HDI) of 0.448. Life expectancy at birth is 64.6 years, and children spend an average of 8.4 years in school. From 2006 to 2017, the Ethiopian economy grew at an annual rate of 10.3% and aspires to be a lower-middle-income country by 2025 [22, 23]. Improving the health and well-being of children and youth is important to achieving this goal. This will boost quality of life, life expectancy, and productivity among Ethiopians, which is needed to ensure the future development of the country. This study aimed to fill the dearth of evidence on physical activity indicators for children and youth in Ethiopia in 2017–2018 and provide for the first time, a report on the physical activity, sedentary behavior, and health-related fitness of our children and youth.

## Methods

### Report Card Team Roles and Responsibilities

Initially, the country Report Committee (RC) leader registered the country to participate in the Global Matrix 3.0 study. The RC leader then established a multidisciplinary research working group including those with prior experience in the area of physical activity and health. The RC leader acted as main country contact and linked the research working group with AHKGA. The Ethiopian RC research working group contained four members (one RC leader, one principal investigator, and two others). The main roles and responsibilities of the RC leader were to provide leadership, coordination, and management of the Global Matrix 3.0 Ethiopian project including the daily activities of the team and liaison with external stakeholders as required. This included developing strategies to collect and analyze the data and dissemination of the findings. The principal investigator focused on the identification of the research question, data sources, data collection tools, synthesizing the evidence, and supporting the research team. Other research working group members focused on searching and summarizing the available data. A mentor from the AHKGA was assigned to provide support to the team. Monthly e-blasts were provided from the AHKGA Executive which outlined the detailed steps and scheduled time for the development of Report Card.

### Report Card Indicators

The ten physical activity indicators for children and youth (overall physical activity, organized sport participation, active play, active transportation, sedentary behaviors, physical fitness, family and peers, schools, community and environment, and government) were used to develop Ethiopia’s Report Card. The AHKGA has a standard grading system for all indicators. The grading system was done through a harmonized process and the standard grading rubric of the Global Matrix 3.0 study ((A = ≥ 80%, B = 60%–79%, C = 40%–59%, D = 20%–39%, F = < 20%, INC = incomplete data). More detailed information is shown in Table 1. The country RC team assigned the grades for each indicator and sent these to the AHKGA scientific committee for evaluation, justification, and audit. Audited grades were converted into interval variables to compute an overall grade [6]. This overall grade revealed how successful Ethiopia was performing in the all indicators of physical activity for children and youth.

**Table 1:** Overview of data sources used in the development of Ethiopia’s 2018 Report Card

Study name	*Global Matrix 3.0 initiative, Ethiopia study (2017–2018)*		
Indicator	Benchmarks	Data Source	Year of publication
*Overall physical activity*	% of children and youth who meet the WHO recommended physical activity and accumulate a minimum of 60 min moderate- to- vigorous-intensity physical activity on a daily basis	[7]	2010
*Organized sport participation*	% of children and youth who engaged in organized sport/physical activity programs	[24]	1998
*Active play*	% of children and youth who participated in unstructured active play for several hours a day	[6, 13]	2018
*Active transportation*	% of children and youth who actively travel to and from places including school, friend’s house, mall, and park	[6, 13]	2018
*Sedentary behaviors*	% of children and youth who achieve the Canadian Sedentary Behaviour guidelines, no more than 2 h of screen time in a day	[6, 13, 14]	2018
*Physical fitness*	% of children and youth who fulfill criterion-referenced standards for muscular strength, endurance, and flexibility	[n/a]	-
*Family and peers*	% of children and youth who encouraged and get support from their family’s members and friends to be physically active	[6, 13]	2018
*School*	% of children and youth who have regular access to facilities and equipment that support physical activity including outdoor play areas, sporting fields, multipurpose space for physical activity, and gymnasium in school	[6, 13, 24]	2018, 1998
*Community and environment*	% of children and youth who living in a safe neighborhood where they have access to infrastructure like sidewalks available to them in their community	[6, 13]	2018
*Government*	Evidence of policy existence, leadership, commitment, and allocating resources for the implementation of physical activity interventions for all children and youth	[24]	1998

### Data Sources

Ethiopia’s 2018 Report Card was developed for the first time based on the latest available data sources. The main sources of data for this study were electronic search databases (PubMed, Google Scholar, Science Direct, Cochrane library, and WHO Hinari) and manual search strategies. There was very limited information on physical activity among children in Ethiopia. Children and youth aged 5–17 years in Ethiopia were included in this study. Data were collected from December 2017 to April 2018. Data were retrieved from published literature, government policy documents [24], and an expert interview panel. This panel comprised experts from several disciplines (public health, sport science, physiotherapy, and sport sector leaders). Each member of panel gave a grade for indicators where no evidence was available. There was then debate on grades mentioned until consensus was reached and the average grade was taken as a final grade. Data were analyzed using the harmonized process and standard grading rubric [6].

### Benchmarks and Consensus

The benchmark for all indicators was the WHO physical activity guideline for children aged from 5–17 years [7] and the Canadian 24-Hour Movement Guidelines for Children and Youth [25]. Detailed explanations of these study methods were presented in Table 1.

## Results

### Physical Activity Indicators for Children and Youth in Ethiopia

This study revealed that there was limited evidence on physical activity indicators for children and youth in Ethiopia. Even though the evidence was limited, major efforts were made to develop Ethiopia’s first Report Card. The highest score was obtained for the “active play” indicator. A detail explanation on each indicator is presented in Table 2.

**Table 2:** Grading and interpretation of the ten core indicators of physical activity among children and youth in Ethiopia, 2018

Indicator	Grade	Grading scheme	Interpretation
Overall physical activity	D	We are succeeding with less than half but some children and youth (27%–33%)	Majority of Ethiopian children and youth take part in home chores and light work every day for family help. Our team estimated that about 28% of children and youth (17% urban & 39% rural) meet 60 min of moderate physical activity every day
Organized sport participations	C	We are succeeding with about half of children and youth (47%–53%)	Almost 50% of children and youth are participating in school athletics, handball, volleyball, and football competitions at all levels several times in a year
Active play	B	We are succeeding with well over half of children and youth (67%–73%)	Experts estimated that 71% of children and youth involved in active play for a minimum of 2 h a day before, during, and after school
Active transportation	C	We are succeeding with about half of children and youth (47%–53%)	Approximately 48% of children and youth (31% in urban and 65% rural) are walking to and from school
Sedentary behaviors	F	We are succeeding with very few children and youth (< 20%)	About 13% of children and youth spend on screen time such as a mobile game, play station, and TV views for no more than 2 h per day
Physical fitness	INC	Incomplete-insufficient or inadequate information to assign a grade	There is no adequate information in the country to assign a grade for this indicator
Family and peers	F	We are succeeding with very few children and youth (< 20%)	Approximately 14% of children and youth are encouraged and get support from their family members like buying bike, handball, or football for their child to move
Schools	D	We are succeeding with less than half but some children and youth (27%–33%)	Our team estimated that 32% of schools in the country have access to infrastructures like a sports field, outdoor playground, and multipurpose spaces for physical activity
Community and environment	F	We are succeeding with very few children and youth (<20%)	Approximately 8% of children and youth live in an environment with accessible infrastructure for physical activity such as sidewalks and parks
Government	D	We are succeeding with less than half but some children and youth (27%–33%)	Policy exists regarding physical activity in the country’s non-communicable disease (NCD) agenda. However, it is not implemented yet

### Overall Physical Activity: D

The overall physical activity indicator is one of the ten core indicators. This indicator aimed to measure the percentage of children and youth who met the WHO recommendation of 60 min of moderate- to vigorous-intensity physical activity a day. There was scant evidence on this indicator in Ethiopia. As a result, the RC team estimated an approximate figure using an expert panel debate and consensus method. The result was that around 28% of children and youth achieved this recommendation. This equated to a “D” according to the AHKGA grading rubric. In Ethiopia, the majority of children and youth are active through home chores and other light work-related activities to help their families.

### Organized Sport Participation: C

This indicator refers to the percentage of children and youth who participated in organized sports programs in Ethiopia. Our RC team determined from their observation and country experience in this area that almost 50% of children and youth in Ethiopia participate in school athletics, handball, volleyball, football, and other sports competitions at all levels for several times in a year. Hence, this indicator was graded as a “C.”

### Active Play: B

This indicator scored the highest grade among ten core indicators. This was obtained by determining the percentage of children and youth who engaged in unstructured physical activity (active play) at any intensity for more than 2 h a day in Ethiopia. The RC team found limited evidence on this indicator. The team estimated the average figure as about 71% of children and youth who might be involved in active play for a minimum of 2 h a day before, during, and after school in Ethiopia. This indicator was graded a “B.”

### Active Transportation: C

This refers to the percentage of children and youth who use active transportation to get to and from places in the form of walking and biking. About 48% of children and youth walk to and from school in Ethiopia. The indicator was scored at grade “C.”

### Sedentary Behaviors: F

This was determined by the percentage of children and youth who engaged in 2 h or less of sedentary screen time a day in Ethiopia. It was estimated that about 13% of children and youth spend less that this amount of time using electronic media for recreational purposes. The indicator was scored a grade “F.”

### Physical Fitness: INC

This indicator was determined by the percentage of children and youth who meet criterion-referenced standards for cardiorespiratory fitness, muscular strength, and endurance in Ethiopia. There was no adequate information in the country to assign a grade for this indicator. Thus, the grade for this indicator was incomplete “INC.”

### Family and Peers: F

This indicator was determined by the percentage of children and youth who get support from their friends, peers, and families to be physically active in Ethiopia. Our experts determined that about 14% of children and youth are encouraged and get support from their family members. This is typically in the form of buying a bike, handball, or football to help their child to move more. The indicator was graded as “F.”

### Schools: D

This indicator was determined by the percentage of schools with active policies and infrastructures that support physical activity participation with trained physical education specialists in the school. Our team estimated that 32% of schools in Ethiopia have access to infrastructure like a sports field, outdoor playground, and multipurpose spaces for physical activity. The indicator was grade as “D.”

### Community and Environment: F

This indicator was determined by the percentage of communities/municipalities that create opportunities for physical activity among children and youth in Ethiopia. Approximately 8% of children and youth live in an environment with accessible infrastructure for physical activity such as sidewalks and parks. The indicator was graded as “F.”

### Government: D

This refers to the existence of government policy evidence that allocates resources to support and implement physical activity initiative for children and youth. There is policy existence for physical activity in the country with non-communicable disease (NCD) agenda. However, there are no allocated resources yet to implement and adapt for children and youth in Ethiopia. The indicator was graded as “D.”

## Discussion

This study revealed a gap in physical activity indicators for children and youth in Ethiopia. Among the ten core indicators, the country scored three grade “Ds” and three grade “Fs.” Two indicators were graded as a “C” and one as a “B.” One indicator was graded incomplete (INC). This indicated a dearth of evidence in physical activity policy, research, and surveillance in Ethiopia. Caution should be given while interpreting these findings since grades were largely informed by the expert panel’s estimation when data for indicators were not available. Nevertheless, our study used standard methods that provide an initial evaluation of physical activity indicators for children and youth in Ethiopia [4, 6].

The average physical activity grade for Ethiopia is “D.” Our finding was comparable with the findings reported from Chile, Ecuador, India, Lebanon, the USA, Uruguay, and Venezuela [26–32]. On the other hand, our finding was lower than those reported for the majority of countries that participated in the Global Matrix 3. Our grade was greater than the results of reported from China [33], which scored grade D [6]. The reason for this finding is likely to be a lack of attention given to physical activity among children and youth in many countries including Ethiopia. Many people assume that children in Ethiopia are highly active and that there are other more pressing public health priorities.

The overall physical activity indicator for children and youth in Ethiopia was low, scored as a grade “D.” This showed that there is a low percentage of children and youth who are participating in the recommended amount of physical activity on a daily basis. This finding was consistent with the findings reported from Brazil, Czech Republic, Ecuador, Finland, France, Guernsey, India, Lebanon, Portugal, Qatar, Spain, Uruguay, and Venezuela [27–29, 31, 32, 34–41]. It was higher than study findings of 18 participating countries [6] including the USA, Australia, Germany, Japan, and China [30, 33, 42–44]. The possible reason might be due to the global concern of physical inactivity regardless of the income status of the country [6].

Ethiopia scored a medium grade, “C” for organized sport participation. Our result was similar to the study reported from Colombia, Estonia, Hong Kong, Lithuania, Mexico, South Korea, and the USA [29, 44–50]. Our finding was lower than 21 other countries in the Global Matrix 3.0 [6]. This variation might be as a result of how countries approach school-based sport competition programs. For instance, there are school athletics, short distance running, handball, volleyball, and football competitions available several times every year in Ethiopia that provide opportunities for children and youth to participate.

Ethiopia achieved the highest grade for the active play indicator, a “B.” Our result was similar to the finding reported from the Netherlands [51]. Our finding was higher than 47 other countries in the Global Matrix 3.0 [6]. This high result is largely due to less accessibility to electronic media, especially in rural areas as compared with high-income countries. In Ethiopia, children and youth are the age groups that mostly engage in unstructured active play for more than 2 h a day before, during, and after school.

Our find reported a medium grade for the active transportation indicator, “C.” This is comparable with results reported from Botswana, Brazil, Poland, Scotland, Slovenia, South Africa, Sweden, Thailand, and Uruguay [31, 34, 52–58]. Our finding was lower than the results of 19 other countries [6]. The difference might be due to environment factors. In Ethiopia, there are limited spaces for walking and cycling for recreation. However, most children and youth engaged in active walking to and from school.

Our results showed that sedentary behavior indicator scored the least grade, “F.” This meant that the majority of children and youth in Ethiopia spent more time in a sedentary lifestyle. Our finding was consistent with the study reported from China, Estonia, Scotland, and Wales [33, 46, 54, 59]. However, our finding was higher than the results reported from Ghana, South Africa, and Venezuela [32, 56, 60]. This might be due to lifestyle and environmental differences among countries. In Ethiopia, many children in urban areas spend their time watching television, whereas in rural areas there is less access to electronic media which provides greater opportunity for physical activity [28].

Our research group was unable to grade the physical fitness indicator due to a lack of available evidence. This was similar to 26 other countries including Zimbabwe [61]. The reason for this might be a lack of evidence on research to support this indicator in many countries.

The family and peers indicator was scored as a grade “F” in Ethiopia. This finding was comparable with the report from Chile, Ecuador, and Ghana [26, 27, 60]. It was higher than results reported from 21 countries including Zimbabwe [61]. The possible reason might be a lack of family and peer encouragement and support from physically inactive people in Ethiopia.

The school indicator was graded a “D” in Ethiopia. Our finding was comparable with the study reported from Chile, Colombia, Ghana, and Lebanon [26, 29, 44, 60]. Our study was higher than results reported from South Africa, United Arab Emirates, the USA, Bangladesh, Ecuador, Guernsey, India, Nepal, Scotland, Venezuela, and Wales [27, 28, 30, 32, 38, 54, 56, 59, 62–64]. The possible reason might be as a result of environmental factors and limited school-based physical activity programs in Ethiopia.

Ethiopia’s grade for the community and environment indicator was “F.” Our finding was similar to the grade reported in China [34]. However, our grade was lower than that reported from 34 other countries [6]. The reason might be as a result of less attention from the government toward the need for accessible built environments to support physical activity such as sidewalks and parks.

The government indicator was graded a “D” in Ethiopia. This is consistent with the grade reported from Australia, Ghana, Guernsey, India, Jersey, South Korea, and Uruguay [28, 31, 38, 42, 50, 60, 65]. Our finding was higher than the grade reported from China, Venezuela, Bulgaria, Ecuador, England, Germany, Nepal, Netherlands, Spain, and the USA [6, 27, 30, 32, 33, 41, 43, 50, 64, 66, 67]. The reason might be due to a low level of political commitment from the country’s leaders toward physical activity.

## Conclusion

This study revealed a number of gaps in physical activity indicators for children and youth in Ethiopia. The results showed that Ethiopian’s children and youth have received low grades for majority of physical activity indicators. Therefore, urgent actions need to be taken by government, policymakers, researchers, and key stakeholders to address the following suggested priority areas:

- Develop and implement a national physical activity and sedentary behavior surveillance system.
- Formulate policy and allocate resources to implement interventions to promote physical activity and reduce sedentary behavior, using the WHO “best-buys” for physical activity promotion.
- Developing a national physical activity plan for children and youth based on principles from the WHO Global Action Plan for Physical Activity (GAPPA).

## Data Availability

Not applicable since this manuscript does not contain any data

## Abbreviations

AHKGA: Active Healthy Kids Global Alliance
GAPPA: Global Action Plan for Physical Activity
HDI: Human Development Index
INC: Incomplete grade
NCDs: Non-communicable diseases
RC: Report card
WHO: World Health Organization
